# Prevalence of hepatitis D virus among HBsAg-positive individuals, 2015-2016: Azar cohort study

**DOI:** 10.15171/hpp.2020.07

**Published:** 2020-01-28

**Authors:** Ali Asghar Pouri, Morteza Ghojazadeh, Babak Baiaz, Fatemeh Soghra Hamzavi, Behrouz Pourasghari, Mohammad Hossein Somi

**Affiliations:** ^1^Liver and Gastrointestinal Diseases Research Center, Tabriz University of Medical Sciences, Tabriz, Iran; ^2^Laborathory Department Imam Reza Hospital, Tabriz University of Medical Sciences, Tabriz, Iran

**Keywords:** Hepatitis D, Hepatitis B, Prevalence, Cohort Studies, Iran

## Abstract

**Background:** Hepatitis D virus (HDV) is a defective RNA pathogen that requires the presence of the hepatitis B virus (HBV) for infection. Middle East countries are endemic areas for HDV infection. So, it is important to estimate the prevalence of HDV in these countries. This study aimed to estimate the prevalence of HDV in HBsAg positive patients participated in Azar cohort study, North-west of Iran.

**Methods:** In this cross-sectional study, out of 4949 participants of the Azar cohort study, 51 HBsAg positive patients were selected. Five participants did not consent to HDV testing. The presence of anti-HDV IgG was checked in 46 patients (13 chronic hepatitis B and 33 inactive chronic hepatitis B) using enzyme-linked immunosorbent assay (ELISA) kit. The serum level of liver enzymes was measured and a questionnaire about risk factors was completed.

**Results:** In this study, the mean age of HBsAg positive patients was 50.06 (SD 9.14) years and 41.3% were female. Only one out of 46 patients was positive for HDV infection. Thus, the prevalence of HDV infection among hepatitis B virus surface antigen (HBsAg) positive patients was 2.17% (95% CI: 0.1-11.5). The positive anti-HDV patient was in the inactive chronic hepatitis B state and she had a history of hospitalization and dental procedures.

**Conclusion:** The results showed that the prevalence of HDV infection in HBsAg positive patients was 2.1% that was lower than the reported prevalence in many other regions of Iran. Health policymakers and healthcare providers should design coherent and orderly epidemiological studies for planning and monitoring HDV infection.

## Introduction

Hepatitis B virus (HBV) infection is one of the most important health problems in the world, especially in developing countries, and causes more than 780 000 deaths annually especially due to complications of liver cirrhosis namely, liver failure and hepatocellular cancer (HCC).^1^ It is estimated that approximately 5% of HBsAg positive people are infected with hepatitis D virus (HDV), and about 15 million people worldwide are chronically co-infected with HBV and HDV.^2^ HDV is an incomplete and defective RNA virus that requires the HBsAg HBV to replicate.^3^ It has been shown that coexistent infection with HDV accelerates the progression of hepatitis B to cirrhosis in 70%-90% of the people, but the lesion is seen in less than 5% of patients with acute HBV without coinfection with the HDV.^4^

Since HDV infection depends on the prevalence of HBV, the prevalence of HDV infection differs in different countries.^5^ Generally, there are two epidemiological patterns of HDV infection. The areas with high prevalence include the Mediterranean, West Africa, Middle East, Central and Northern Asia, and the areas with a low prevalence include South Africa, Northern Europe, North America, and Eastern Asia.^6^ It has been reported that Middle East countries are endemic areas for HDV infection. For instance, the prevalence of HDV was 16.6%-88.8% in different regions of Pakistan^7^ and less than 5% in the western region of Turkey and more than 27% in southeastern Turkey.^8^

There are limited studies conducted on the prevalence of HDV in Iran. According to reports, the prevalence of hepatitis D differs across the provinces; the highest prevalence was reported in Hamedan (17.3%),^9^ followed by Kerman (16.75%)^10^ and Fars (9.7%).^11^ The lowest prevalence was reported from Babol (2%),^12^ Qom (2%)^13^ and Birjand (1.2%).^14^

Two previous studies also investigated the frequency of HDV infection in patients with hepatitis B in East-Azerbaijan. Torabi et al studied the frequency of HDV in HbsAg positive patients and blood donors in Tabriz in 2003 and showed that 6.15% of studied cases were positive for anti-HDV.^15^ In another study, Seifi and Ghannad reported that the prevalence of HDV in HbsAg positive patients was 6.01% in Tabriz in 2006.^16^ One study was also conducted in Tehran and Tabriz in 2007 on asymptomatic and symptomatic HBsAg positive patients and reported the prevalence of 9.3%.^17^ Considering the decreasing trend of hepatitis B prevalence in this region in recent years,^18^ it is important to have updated information about the prevalence of HDV for planning of the prevention programs. To the best of our knowledge, there is no updated data in this regard in North-west of Iran and also there is no study in Shabestar county in East Azerbaijan (Azar cohort area). So, this study was conducted to estimate the prevalence of hepatitis D co-infection with hepatitis B in the Azar cohort area.

## Materials and Methods

Data from this cross-sectional study was obtained from the Azar cohort study, in Khameneh in East-Azerbaijan province (North West of Iran). This cohort is part of the large Persian cohort study (Prospective Epidemiological Research Studies of Iranian Adults).^19^ Azar cohort study was conducted on 5000 adult population (age >35 years old) of Khameneh and vicinity in a time period between 2015 and 2016. The sampling method and inclusion criteria of the Azar cohort study were described in detail in a previous study.^20^

For the aim of the present study, from the Azar cohort population, we included the participants with HBsAg positivity. The HbsAg and HbcAb positivity were checked using the ELFA method (Enzyme-Linked Fluorescent Assay; Vidas^®^ HBsAg, Biomerieux, France; Vidas^®^ HBcAb, Biomeriux, France) at the Azar cohort laboratory. In 51 cases, HBsAg was positive. Out of this number, 46 patients (13 patients with chronic hepatitis B and 33 patients with inactive chronic hepatitis B) were enrolled and 5 subjects were excluded due to their unwillingness to participate in the next phase of the study. Anti-HDV IgG was measured by ELISA test (DIA.PRO, Italy kit; less than 0.9 was considered negative and above 1.1 was positive). In addition, the serum concentration of aspartate aminotransferase (AST), alanine aminotransferase (ALT), gamma-glutamyl transferase (GGT) and alkaline phosphatase (ALP) were measured using the Pars Azmon kits (Tehran, Iran) by photometric method and autoanalyzer biochemistry (Mindray, model Bs380, China ) at the Azar Cohort Laboratory. A questionnaire containing some demographic information and risk factors for hepatitis such as history of transfusion, surgery, hospitalization, dental procedures, traveling abroad and drug abuse was completed.

Data were analyzed using (SPSS, version 19; SPSS Inc., Chicago, IL, USA). Data were presented as Mean (SD) for quantitative variables and frequency (%) for qualitative variables.

## Results

This study determined the prevalence of hepatitis D in the Azar cohort population. In this study, out of 46 patients with HBsAg positive (28.3% chronic hepatitis B and 71.7% inactive chronic hepatitis B) who consent to HDV testing, 27 (58.7%) were male and 19 (41.3%) were female (Table 1). The mean age of patients was 50.06 ± 9.14 years. The results showed that only one out of 46 patients was positive for HDV infection. The prevalence of HDV infection among HBsAg positive patients was 2.17% (95% CI: 0.1-11.5) and among the entire cohort population was 0.02 % (95% CI: 0.001-0.1).

The anti-HDV positive patient was in the inactive chronic hepatitis B state and married, 58-year-old female who lived in Shabestar county. She was overweight (BMI=27.3 kg/m^2^). She tested negative for HBcAb, HBsAb and her liver enzymes level were normal (AST=19 IU/L, ALT=22 IU/L, ALP=249 IU/L, GGT=18 IU/L). She suffered from joint pain and had a history of hospitalization and dental procedures; she had no history of surgery, blood transfusion, traveling abroad, addiction, icterus, weight loss and alcohol drinking or smoking.

## Discussion

HDV in hepatitis B patients causes liver failure or chronic hepatitis, and increase the risk of HCC compared to HBV mono-infection.^21^ So, determining its prevalence could be useful for planning of the prevention programs. In this epidemiological study, the prevalence of HDV infection was investigated among the HBsAg positive patients who participated in the Azar cohort study. According to the result, the prevalence of HDV infection among HBsAg positive patients was 2.17% and the prevalence of HDV infection in the entire cohort population was 0.02%. The prevalence of HDV in the present study was similar to reported prevalence in a meta-analysis in Iran,^22^ Isfahan (2.9%)^23^ and Qom (2%).^13^ However, it is lower than the reported prevalence rate in Hamadan (17.3%),^9^ Ahvaz (11.5%),^24^ Birjand (3.1%),^25^ and Tabriz and Tehran (9.3%).^17^ The observed difference in the prevalence of HDV infection may be attributable to the differences in the age distribution of studied population, sampling source, geographical region and time of the study. Esmaeilzadeh et al showed that the prevalence of HDV varied according to the sampling source; the prevalence of HDV was higher among HIV patients (19.7%) compared to other population.^5^Moreover, the age of participants could also affect the prevalence of HDV. Previous studies showed a higher prevalence of HDV in patients aged over 40 years old.^17^ In addition, considering that HDV is dependent on HBV, the differences in HBV prevalence in different regions of Iran could justify the observed differences in HDV infection in different studies. Moreover, the higher prevalence of HDV in some regions of Iran such as Kerman may be due to the high immigration of foreigners from Afghanistan and Pakistan.^10^ However, this phenomenon does not exist in our region. In addition, considering that HDV is more prevalent in a population of low socioeconomic status,^26^ it is postulated that the lower prevalence of HDV in our region may be due to the higher health and socioeconomic status of the population.

Compared with earlier studies in North-west of Iran,^15,16^ the prevalence of HDV was lower in the present study that may be due to the decreasing prevalence rate of hepatitis B in this region. In a study by Somi et al, the significant downward trend of HBV was reported in East Azerbaijan from 2011 to 2016.^18^

Compared with nearby countries, the prevalence of HDV was lower in our region. In recent studies, the prevalence of HDV infection in HBsAg positive patients was reported to be 9.6% in Turkey and 6.6% in Iraq.^27,28^ The differences in HDV prevalence between different countries may be related to variation in the prevalence of risk factors. Like HBV, transmissions from blood products, intravenous drug users, sexual behaviors and maternal-child transmission are the most prevalent rout of transmissions for HDV.^29-31^In Iran, all blood donors were screened for hepatitis infection from 1996^32^ and high‐risk donors were eliminated; therefore, this transmission route was excluded. Furthermore, screening of pregnant women for hepatitis infection decreases the vertical transmission of hepatitis virus.^33^

In our study, the anti-HDV positive patient was a 58-year-old woman. Somi et al showed that the risk of HDV increases significantly after the age of 40.^17^ In another study in Birjand, a positive case of anti-HDV was a 55-year-old woman.^14^ Moreover, in the present study, the anti-HDV positive case had a history of hospitalization and dental procedures. It has been shown that HDV is transmitted chiefly through parenteral exposure in the same way as HBV.^34^

The results of the present study should be interpreted considering the limitations of the study including the low sample size. Considering that only one HDV positive patient was identified in the present study, it was not possible to compare the prevalence of HDV in different patients in terms of risk factors for hepatitis, sex and age.

## Conclusion

The results of the present study showed that the prevalence of HDV infection in HBsAg positive patients was 2.1% that was lower than the reported prevalence in many other regions of Iran. Moreover, this rate was lower than the previously reported prevalence in the North-West of Iran. Since the prevalence of HDV in different geographical areas is changing over time, it is suggested that health policymakers and healthcare providers design coherent and orderly epidemiological studies for planning and monitoring HDV infection as well as HBV prevention programs among high-risk groups. From the research point of view, for assessing the risk factors of HDV infection larger studies are needed to conduct in the North-West of Iran. Future molecular and genotypic distribution studies are also suggested.

## Ethical approval

This research was approved by the Regional Ethics Committee of Tabriz University of medical Sciences (IR.TBZMED.REC.1395.313) and information was kept secret.

## Competing interests

The authors declare that they have no competing interests.

## Funding

This study was granted by Liver and Gastrointestinal Diseases Research Center, Tabriz University of Medical Sciences.

## Authors’ contributions

MHS was responsible for the conception and design of the study. AAP, BB, FSH and BP were responsible for the data acquisition and laboratory analysis. MG was responsible for data analysis and interpretation. AAP drafted the manuscript; MG, BB, FSH, BP and MHS revised and commented on the draft, and all authors read and approved the final version of the manuscript.

## Acknowledgments

The authors are grateful for the financial support of the liver and gastrointestinal diseases research center, Tabriz University of Medical Sciences. The authors also are deeply indebted to all subjects who participated in this study. We appreciate the contribution of the investigators and the staff of the Azar cohort study. We thank the close collaboration of the Shabestar health center. In addition, we would like to thank the Persian cohort study staff for their technical support. This article has been extracted from a PhD thesis (No. 1395.313).

**Table 1:** Prevalence of HBsAg according to some risk factors and some demographic characteristics

**Variable**	**Level**	**HBsAg Positive ( N=46)**
Sex	Male	27 (58.7)
	Female	19 (41.3)
Marital status	Single	1 (2.2)
	Married	45 (97.8)
Surgery history	Yes	24 (52.2)
	No	22 (47.8)
Hospitalization history	Yes	35 (76.1)
	No	11 (23.9)
Dental procedures history	Yes	18 (39.2)
	No	28 (60.8)
Transfusion history	Yes	4 (8.7)
	No	42 (91.3)
Traveling abroad	Yes	25 (54.4)
	No	21 (45.6)
Drug addiction	Yes	2 (4.4)
	No	44 (95.6)

Abbreviation: HBsAg, hepatitis B virus surface antigen. Data are shown as No. (%).
